# SSRI use during acute COVID-19 and risk of Long COVID among patients with depression

**DOI:** 10.1186/s12916-024-03655-x

**Published:** 2024-10-08

**Authors:** Zachary Butzin-Dozier, Yunwen Ji, Sarang Deshpande, Eric Hurwitz, A. Jerrod Anzalone, Jeremy Coyle, Junming Shi, Andrew Mertens, Mark J. van der Laan, John M. Colford, Rena C. Patel, Alan E. Hubbard, the National COVID Cohort Collaborative (N3C) Consortium

**Affiliations:** 1 School of Public Health, University of California, Berkeley, Berkeley, CA USA; 2 University of North Carolina at Chapel Hill, Chapel Hill, NC USA; 3 University of Nebraska Medical Center, Omaha, NE USA; 4 University of Alabama at Birmingham, Birmingham, AL USA

**Keywords:** COVID-19, Long COVID, SSRI

## Abstract

**Background:**

Long COVID, also known as post-acute sequelae of COVID-19 (PASC), is a poorly understood condition with symptoms across a range of biological domains that often have debilitating consequences. Some have recently suggested that lingering SARS-CoV-2 virus particles in the gut may impede serotonin production and that low serotonin may drive many Long COVID symptoms across a range of biological systems. Therefore, selective serotonin reuptake inhibitors (SSRIs), which increase synaptic serotonin availability, may be used to prevent or treat Long COVID. SSRIs are commonly prescribed for depression, therefore restricting a study sample to only include patients with depression can reduce the concern of confounding by indication.

**Methods:**

In an observational sample of electronic health records from patients in the National COVID Cohort Collaborative (N3C) with a COVID-19 diagnosis between September 1, 2021, and December 1, 2022, and a comorbid depressive disorder, the leading indication for SSRI use, we evaluated the relationship between SSRI use during acute COVID-19 and subsequent 12-month risk of Long COVID (defined by ICD-10 code U09.9). We defined SSRI use as a prescription for SSRI medication beginning at least 30 days before acute COVID-19 and not ending before SARS-CoV-2 infection. To minimize bias, we estimated relationships using nonparametric targeted maximum likelihood estimation to aggressively adjust for high-dimensional covariates.

**Results:**

We analyzed a sample (*n* = 302,626) of patients with a diagnosis of a depressive condition before COVID-19 diagnosis, where 100,803 (33%) were using an SSRI. We found that SSRI users had a significantly lower risk of Long COVID compared to nonusers (adjusted causal relative risk 0.92, 95% CI (0.86, 0.99)) and we found a similar relationship comparing new SSRI users (first SSRI prescription 1 to 4 months before acute COVID-19 with no prior history of SSRI use) to nonusers (adjusted causal relative risk 0.89, 95% CI (0.80, 0.98)).

**Conclusions:**

These findings suggest that SSRI use during acute COVID-19 may be protective against Long COVID, supporting the hypothesis that serotonin may be a key mechanistic biomarker of Long COVID.

**Supplementary Information:**

The online version contains supplementary material available at 10.1186/s12916-024-03655-x.

## Background

SARS-CoV-2 infection can have debilitating long-term consequences. Long COVID, also known as post-acute sequelae of COVID-19 (PASC), includes symptoms across a range of biological systems that can occur following SARS-CoV-2 infection. Millions of adults in the United States may be experiencing Long COVID, the majority of whom only experienced mild to moderate COVID-19 [1, 2]. Even though more than 10% of COVID-19 patients develop Long COVID, we have few insights regarding options for treatment and prevention [3]. Insights regarding treatments that may prevent Long COVID are crucial to preventing this condition and understanding its etiology.

**Fig. 1:**
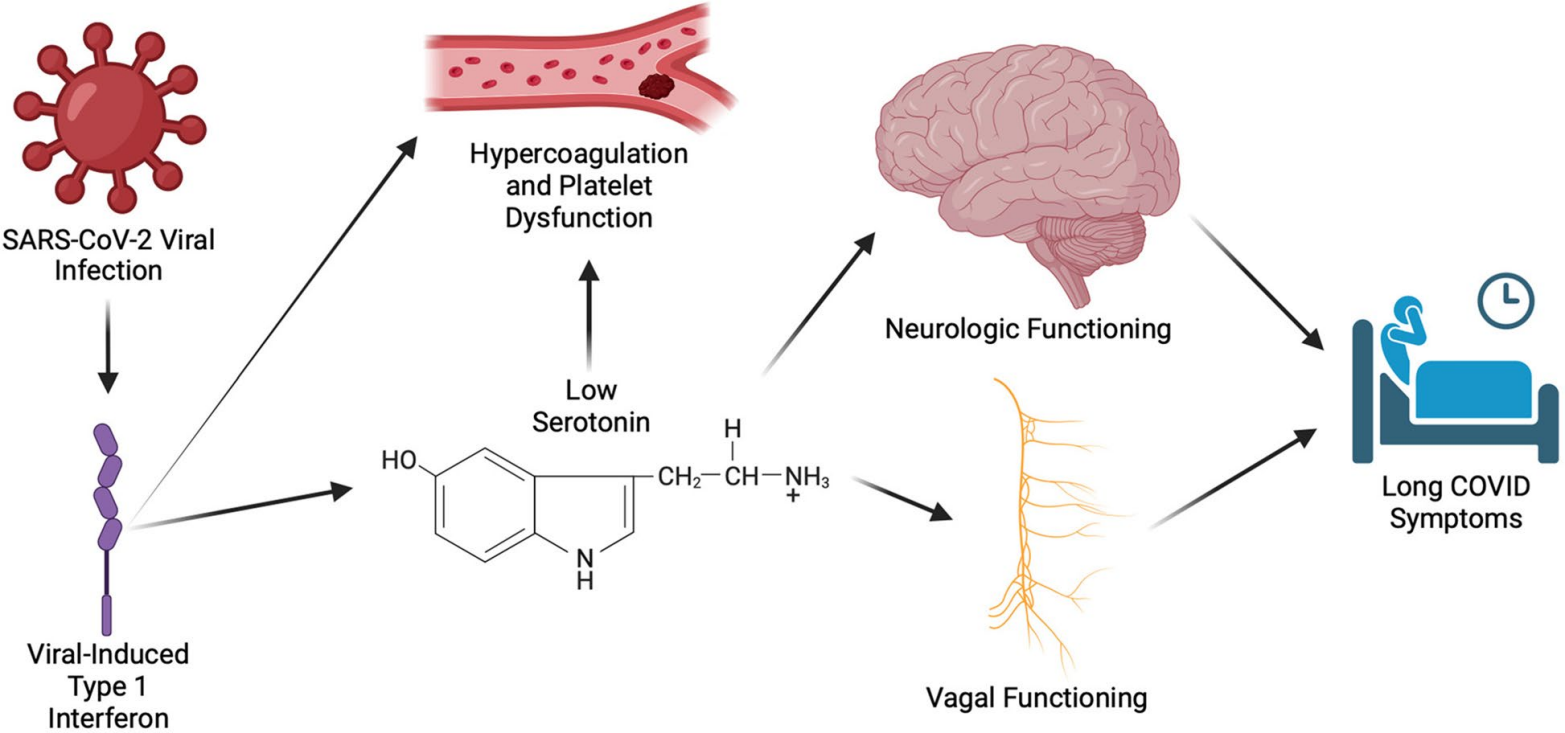
Hypothesized mechanism of the relationship between serotonin and Long COVID [4, 7]

Investigators have hypothesized several biological mechanisms that drive Long COVID and lead to clusters of symptoms. These explanations include [1] persistent COVID-19 viral load, [2] chronic hyperinflammation, [3] platelet and coagulation issues, and [4] central nervous system dysfunction [4, 5]. Previous studies have clustered these symptoms and speculated that these pathways may be distinct disorders caused by different components of acute COVID-19 [6]. On the other hand, recent investigations have highlighted reduced serotonin as a driver of all four of these symptom clusters [4]. A metabolomics investigation found that persistent COVID-19 viral load led to sustained interferon response, decreased tryptophan (a serotonin precursor) uptake, hypercoagulation, and subsequent decrease in serotonin [4]. This peripheral serotonin deficiency leads to reduced vagus nerve activity, which subsequently contributes to decreased hippocampal activity, which can result in memory loss and cognitive dysfunction (Fig. 1) [4].

Selective serotonin reuptake inhibitors (SSRIs) are the first-line medication class used to treat depression. They have high tolerability and are considered safe and effective [8, 9]. SSRI’s mechanism of action is to prevent serotonin reuptake by inhibiting serotonin transporter at the presynaptic axon terminal. The prevention of this reuptake allows for a greater concentration of serotonin in the synaptic cleft that can bind to receptors [8]. Compared with other classes of antidepressants, such as tricyclic antidepressants or monoamine oxidase inhibitors, SSRIs have fewer side effects due to fewer effects on other neurotransmitters and receptors [8]. Given SSRI’s specific targeting of serotonin, it is an ideal candidate to evaluate the role of serotonin in the development of Long COVID.

Several studies have investigated the relationship between SSRI use and acute SARS-CoV-2 infection as well as Long COVID. The TOGETHER trial found that early treatment with the SSRI fluvoxamine improved COVID-19 patient recovery [10]. On the other hand, the COVID-OUT trial found that fluvoxamine treatment during acute COVID-19 did not reduce the cumulative incidence of Long COVID (1.36, 95% CI (0.78–2.34)), although this analysis included a relatively small sample size (334 patients assigned to fluvoxamine) and may have been underpowered [11]. More broadly, previous studies have found that SSRI use may reduce the probability of hospitalization or mortality due to SARS-CoV-2 infection [12, 13]. A 2022 study evaluated the relationship between SSRI use and the predicted PASC and found that SSRI use was associated with 0.75 (95% CI, 0.62, 0.90) times the risk of predicted PASC compared to non-use [14]. While this observational study provided evidence that SSRI use may be protective against Long COVID, this study used predicted PASC diagnosis (via XGBoost machine learning) as its primary endpoint, rather than actual PASC diagnosis. This predicted PASC status did not directly include SSRI use in its prediction, but it did use a myriad of other diagnoses and medications, which may be correlated with SSRI use and may have induced bias. Furthermore, the study used a general sample of SSRI users and nonusers rather than restricting to conditions that may yield SSRI use, leading to the possibility of residual confounding by indication, as a recent study found that personality and psychiatric disorders were associated with Long COVID [15]. In addition, another observational study found that patients experiencing Long COVID experienced improvement in self-reported symptoms following treatment with SSRIs [16].

Several investigators have evaluated the impact of individual types of SSRIs on Long COVID. A multi-system study of the relationship between serotonin and Long COVID hypothesized that fluoxetine may be particularly effective in preventing and treating Long COVID, and they found that treating mice with fluoxetine improved cognitive function and restored tryptophan levels [4]. Furthermore, animal models involving fruit flies have demonstrated that the specific SSRI types fluoxetine, escitalopram, citalopram, and paroxetine may differentially impact serotonin reuptake [17]. A systematic review of studies evaluating the use of fluvoxamine for COVID-19 and Long COVID suggested that baseline use of fluvoxamine may reduce the risk of Long COVID due to the drug’s sigma 1 receptor agonist activity and the role of sigma 1 receptor activity in acute SARS-CoV-2 infection [18]. Observational analyses of human electronic health record (EHR) data did not find a significant difference in the relationship between moderate to high-affinity sigma 1 receptor agonist SSRIs (fluvoxamine, fluoxetine, escitalopram, and citalopram) versus non-high affinity SSRIs (sertraline and paroxetine) in their impact on Long COVID [14].

The purpose of this study is to evaluate the impact of SSRI use during acute COVID-19 on subsequent Long COVID risk. This study evaluates a potential pharmaceutical intervention to prevent Long COVID while testing a hypothesis regarding a mechanistic pathway of Long COVID. Identifying interventions that prevent Long COVID is crucial for clinical applications as well as our understanding of underlying biological mechanisms. Nationally sampled electronic health record (EHR) databases, such as the National COVID Cohort Collaborative (N3C), provide an excellent opportunity to evaluate these hypotheses but require analytic methods that can aggressively adjust for high-dimensional confounders without making bias-inducing parametric assumptions [19–24]. While randomized controlled trials may eventually provide definitive evidence regarding the benefit of SSRI use to prevent or treat Long COVID, observational analyses, using appropriate methods that are designed to leverage the complexity, including missing data, and large sample sizes characteristic of EHR real-world data (RWD) can provide early insights regarding the relationship between SSRI use and Long COVID. Thus, to evaluate the relationship between SSRI use during COVID-19 and Long COVID cumulative incidence, we conducted an observational analysis of individuals in N3C with an acute SARS-CoV-2 infection and comorbid depression diagnosis using a machine-learning-based method targeted to reduce bias due to confounding and missing data (Targeted Machine Learning) [19, 20, 22–24].

## Methods

### Study sample, data source, and study design

Our primary study sample included individuals with a diagnosis of acute SARS-CoV-2 infection between September 1, 2021 (ensuring that all patients were eligible to be diagnosed with PASC during person-time at risk, as PASC ICD-10 code U09.9 was released October 1, 2021) and December 1, 2022, as well as a comorbid diagnosis of a depressive disorder (see concept IDs listed in Additional file 1: Supplemental Table 1) [25, 26]. This sample was drawn from patients in N3C (DUR-80D09B6), which includes 22 million patients from 83 healthcare institutions [21]. N3C provides high-dimensional, longitudinal data on these patients, which enables researchers to conduct evaluations of a wide range of factors related to Long COVID and acute COVID-19 while rigorously adjusting for factors related to medical history and sociodemographic information.

We constructed a retrospective cohort of patients in N3C who were diagnosed with a depressive disorder (depression) before their acute SARS-CoV-2 infection, and we excluded patients with a prior diagnosis of bipolar disorder. As SSRI prescription is often indicated by depression, we restricted our sample to only include those with depression to limit confounding by indication. We evaluated SSRI use (as a time-invariant, binary variable) at the time of acute COVID-19, and we assessed patients’ cumulative incidence of Long COVID (PASC) between 1 and 12 months (i.e., day 31 to 365) following acute SARS-CoV-2 infection, comparing SSRI users to nonusers. We included patients from 80 data partners (contributors of patient data) in N3C. We found that 23% of data partners did not report PASC diagnosis, and 6% of data partners did not report SSRI use in this study sample.

N3C inclusion criteria for identifying COVID-19 patients includes either [1] at last one laboratory diagnostic positive result (either PCR or antigen) or [2] a provider diagnosis (ICD-10-CM U07.1). We used the earliest of the two dates as the index date for SARS-CoV-2 infection [27].

### Key covariates

#### Exposures

We defined the exposure of interest as a binary variable that represents SSRI use (fluoxetine, sertraline, paroxetine, citalopram, escitalopram, fluvoxamine, and vilazodone) during incident COVID-19. We defined SSRI users as patients who began using an SSRI at least 30 days before COVID-19 and continued through acute COVID-19 (binary, time-invariant), and we defined all other patients as nonusers.

#### Outcomes

Our outcome of interest was observed PASC diagnosis, which was defined by ICD code U09.9, between 1 and 12 months following acute SARS-CoV-2 infection [28]. We included observed PASC (U09.9) diagnosis as our outcome of interest, as it provides a standardized metric of Long COVID incidence across diagnostic settings. In contrast, using the predicted probability of PASC diagnosis (e.g., via machine learning methods) may induce bias if the predictions are generated using the same EHR data as the exposures of interest [14]. We ensured that all patients would have 12 months of follow-up by restricting to patients who were diagnosed with COVID-19 between September 1, 2021 (1 month before the implementation of ICD code U09.9) [25] and December 1, 2022, and including PASC diagnosis data within 12 months of SARS-CoV-2 infection (i.e., up to December 1, 2023). We will describe PASC (ICD code U09.9) as “Long COVID” hereafter.

#### Subgroups of interest

We created subgroups of individuals with specific SSRI drug type exposures for SSRIs with a sufficient sample size, which included fluoxetine, sertraline, paroxetine, citalopram, and escitalopram. Vilazodone and fluvoxamine had insufficient sample sizes and, therefore, were excluded from subgroup analyses. We constructed separate models for each SSRI of interest to assess potential effect heterogeneity. Furthermore, we conducted exploratory analyses of potential dose–response relationships by analyzing subgroups defined by SSRI dosage among fluoxetine users, given fluoxetine’s large sample size and hypothesized relationship with Long COVID [14]. Finally, to evaluate the possibility of residual bias due to history of SSRI use, we conducted a subgroup analysis comparing new SSRI users (new prescription for an SSRI between 1 and 4 months before acute COVID-19 and no prior history of SSRI prescription) to SSRI nonusers.

#### Confounders and other covariates

We extracted extensive medical histories from patients in N3C to adjust for a rich history of patient data and thus avoid unmeasured confounding. Our set of baseline covariates included the following: healthcare utilization rate (number of healthcare interactions pre-SARS-CoV-2 infection and healthcare interactions per month before SARS-CoV-2 infection), sex, age at acute SARS-CoV-2 infection, race/ethnicity, common data model format, region of residence, body mass index (BMI), tobacco smoking status, obesity, diabetes, chronic lung disease, heart failure, hypertension, use of systemic corticosteroids, depression severity, anxiety, antipsychotic medication use, benzodiazepine medication use, whether the patient was immunocompromised, and the number of COVID-19 vaccination doses before infection [29]. We defined a healthcare interaction as a single interaction, or cluster of interactions, with a healthcare provider that was associated with a given medical condition, diagnosis, or procedure. We included county-level socioeconomic variables that included the percent of the county with an income level below the poverty line and the county’s social deprivation index score. We also used methods that can minimize bias due to differential monitoring among patients, by including an indicator variable for whether the patient had a documented healthcare interaction between 1 and 12 months following acute SARS-CoV-2 infection (the outcome observation period). For additional covariate information, see Additional file 1: Appendix 1.

#### Negative control outcome

We sought to evaluate a negative control outcome to evaluate the possibility of bias. We evaluated bone fracture diagnosis between 1 and 12 months after acute COVID-19 diagnosis as a negative control outcome.

### Analysis

To accomplish the goals of using nonparametric statistical methods that could adjust for rich, messy patient history and monitoring data, we applied a Targeted Learning approach, which is well-suited for this context of observational analyses of electronic health record data [19, 20, 23, 30]. Traditional parametric analyses make assumptions regarding model form and relationships between covariates, and these assumptions will inevitably be violated in this high-dimensional setting. This potential model misspecification would increase bias and the probability of type 1 error, particularly given our large sample size [19, 20, 23, 30]. On the other hand, Targeted Learning utilizes advances in machine learning and causal inference by capitalizing on the extensive data to minimize bias introduced by arbitrary modeling assumptions, which can result in improper under-adjustment of confounders. In addition, Targeted Learning methods provide robust statistical inference despite data-adaptive, machine learning methods being used to estimate the statistical relationships of interest.

Our goal was to estimate the impact of SSRI use at the time of acute COVID-19 on the probability of developing Long COVID by comparing the predicted distribution of Long COVID under universal (i.e., all patients using SSRIs) versus no use of SSRIs among our target population, patients with a depressive disorder, under a scenario of universal monitoring of patients between 1 and 12 months after acute COVID-19. Our analysis approach first used Super Learner, an ensemble machine learning algorithm, to model Long COVID status given individual covariate status (diagnoses, treatment, demographics, and other history) [31, 32]. Super Learner uses cross-validation to determine the optimal weighting of candidate algorithms to maximize a parameter of interest. Next, we used targeted maximum likelihood estimation to estimate the causal parameter of interest (the risk ratio) comparing Long COVID incidence in the exposed versus unexposed population [19, 20, 23, 30]. Targeted maximum likelihood estimation allows us to generate interpretable measures of association, such as a risk ratio while reducing bias. In addition, targeted maximum likelihood estimation is doubly robust, meaning that it guarantees consistent estimation as long as the outcome regression or propensity score is estimated consistently [19, 20, 23, 30].

As Super Learner guarantees that the ensemble will perform at least as well as the best-performing candidate learner, given sufficient sample size, we sought to include a diverse library of parametric and nonparametric candidate algorithms to ensure optimal performance [31, 32]. We included the following candidate algorithms: generalized linear models (SL.glm), Bayesian Additive Regression Trees (tmle.SL.dbarts2), Generalized Linear models net (SL.glmnet), XGBoost (SL.xgboost), Caret (SL.caret), Caret Recursive Partitioning and Regression Trees (SL.caret.rpart), K Nearest Neighbors (SL.knn), Neural Net (SL.nnet), Random Forest (SL.randomForest), and Recursive Partitioning and Regression Trees (SL.rpart) [31, 32]. We also used cross-validated (cross-fitted) targeted maximum likelihood estimation (TMLE), which avoids overfitting and adds robustness [19, 20, 23, 30].

We applied a *W*, *A*, $\Delta$, $\Delta Y$ data structure, where *W* referred to our baseline confounders and covariates of interest, *A* referred to our exposure of interest, $\Delta$ referred to participant observation during the outcome period (months 1–12), and $\Delta Y$ referred to our observed outcome. If a participant did not have a healthcare interaction during the outcome window (months 1–12 following SARS-CoV-2 infection), which could be due to lack of healthcare engagement or patient death before observation, $\Delta$ would be equal to 0. We defined our causal parameter of interest as $E(Y(\text{1,1})-Y(\text{0,1}))$, where $Y(a,\Delta =1)$ is defined as the counterfactual outcome if SSRI status is set to *A* = *a*, and the person was monitored during the at-risk period ($\Delta =1$). We intervened on $\Delta$ to ensure that all patients were observed (had at least one healthcare visit) during the outcome window (between 1 and 12 months following SARS-CoV-2 infection). We make the assumption that the subset of confounders that are observed for each subject was sufficient to adjust for confounding; operationally, this was done by adding new basis functions for confounders with missing values, which were indicators that the variable was observed, and imputed values for the missing covariate. This allows us to aggressively adjust for confounding and keep observations with missing covariate information (*W*) [24, 29].

#### Sensitivity analyses

In order to evaluate underlying biases in our analysis and data, we conducted a nonparametric sensitivity analysis [33]. This nonparametric sensitivity analysis allows us to compare the theoretical bias that would nullify our results to benchmarks, such as the difference between our observed adjusted estimate and unadjusted estimate, that could explain the magnitude of our observed association. Furthermore, we evaluated the relationship between SSRI use during SARS-CoV-2 infection and bone fracture between 1 and 12 months following SARS-CoV-2 infection as a negative control outcome analysis. We compared our observed, adjusted result to the [1] unadjusted association and [2] the negative control outcome association.

## Results

### Descriptive statistics

We analyzed EHR data from a sample of 302,626 patients who were diagnosed with a depressive disorder before COVID-19 diagnosis. Among these patients, 100,803 (33%) were using an SSRI at the time of SARS-CoV-2 infection and 201,823 (67%) were not (Table 1, see Additional file 1: Supplemental Fig. 1). We found that SSRI users generally had a greater burden of disease and more markers of poor health than SSRI nonusers. Among SSRI users, 17% were morbidly obese compared to 16% of nonusers, 14% had experienced heart failure compared to 11% of nonusers, 34% had experienced lung disease compared to 31% of nonusers, and 64% used systemic corticosteroids compared to 54% of nonusers. We found that 27% of both groups were smokers. We observed that 47% of SSRI users were diagnosed with an anxiety-related condition and 17% were prescribed benzodiazepines, while 60% of nonusers were diagnosed with an anxiety-related condition and 22% were prescribed benzodiazepines. We also found that 8% of SSRI users had severe major depressive disorder, compared with 6% of non-SSRI users. SSRI users had a healthcare utilization rate of 3.0 healthcare interactions per month, while nonusers had 2.3 healthcare interactions per month. We found that 30% of SSRI users had at least one dose of a COVID-19 vaccination, and 34% of nonusers had at least one vaccination dose.

**Table 1:** Patient characteristics

Characteristic	Value	SSRI Users Count/Mean	SSRI Users Percentage/Std Dev	Non SSRI Users Count/Mean	Non SSRI Users Percentage/Std Dev
Total		101016	33.3	202354	66.7
Sex	FEMALE	74823	74	142930	70.8
	MALE	25799	25.6	58975	29
	Other/missing	394	0.4	449	0.2
Age	(0.0, 17.0]	3437	3.4	9063	4.5
	(17.0, 49.0]	44842	44.4	91366	45.2
	(49.0, 70.0]	35076	34.7	70885	35
	(70.0, 107.0]	17643	17.5	30884	15.3
Ethnicity	White Non-Hispanic	73703	73	130036	64.3
	Black or African American Non-Hispanic	10860	10.8	27919	13.8
	Asian Non-Hispanic	1704	1.7	5492	2.7
	Other Non-Hispanic/Unknown	1156	1.1	3414	1.7
	Hispanic or Latino Any Race	8287	8.2	24721	12.2
	Unknown	5306	5.3	10772	5.3
BMI	(0.0, 25.0]	14842	14.7	35932	17.8
	(25.0, 30.0]	19876	19.7	44054	21.8
	(30.0, 35.0]	19123	18.9	38443	19
	(35.0, 40.0]	13868	13.7	25611	12.7
	(40.0, 100.0]	17569	17.4	31343	15.5
	Missing	15738	15.6	26971	13.3
Medical Conditions	Systemic Corticosteroid Use	64516	63.9	108821	53.8
	Antipsychotic Medication Use	4767	4.7	15761	7.8
	Benzodiazepine Use	17201	17	44858	22.2
	Lung Disease	34308	34	62640	31
	Diabetes	26493	26.2	47131	23.3
	Other Immunocompromised	14667	14.5	28194	13.9
	Smoking	26746	26.5	54079	26.7
	Heart Failure	13712	13.6	21722	10.7
	Hypertension	51323	50.8	93227	46.1
	Anxiety	47382	46.9	121307	59.9
Depression Severity	Mild major depression	11593	11.5	18514	9.1
	Severe major depression	8493	8.4	12306	6.1
	Unknown	80929	80.1	171532	84.8
Medical Utilization	Medical Visits per Month Prior to COVID-19	3.02	3.53	2.25	2.96
	Number of COVID-19 Vaccinations	0.72	1.18	0.77	1.2
Socioeconomic Factors	Percent of County Below Poverty Line	14.9	5.09	15.09	5.04
	County Social Deprivation Index	43.43	27.07	49.12	27.39
COVID-19 factors	Number of COVID-19 Vaccinations	0.72	1.18	0.77	1.2
	At lease one dose of vaccine	37989	30.4	129804	34
	Covid Associated Hospitalization	27927	22.4	96479	25.3
	Long COVID diagnosis	1735	1.7	3337	1.6

### Relationship between SSRI use and long COVID

**Fig. 2:**
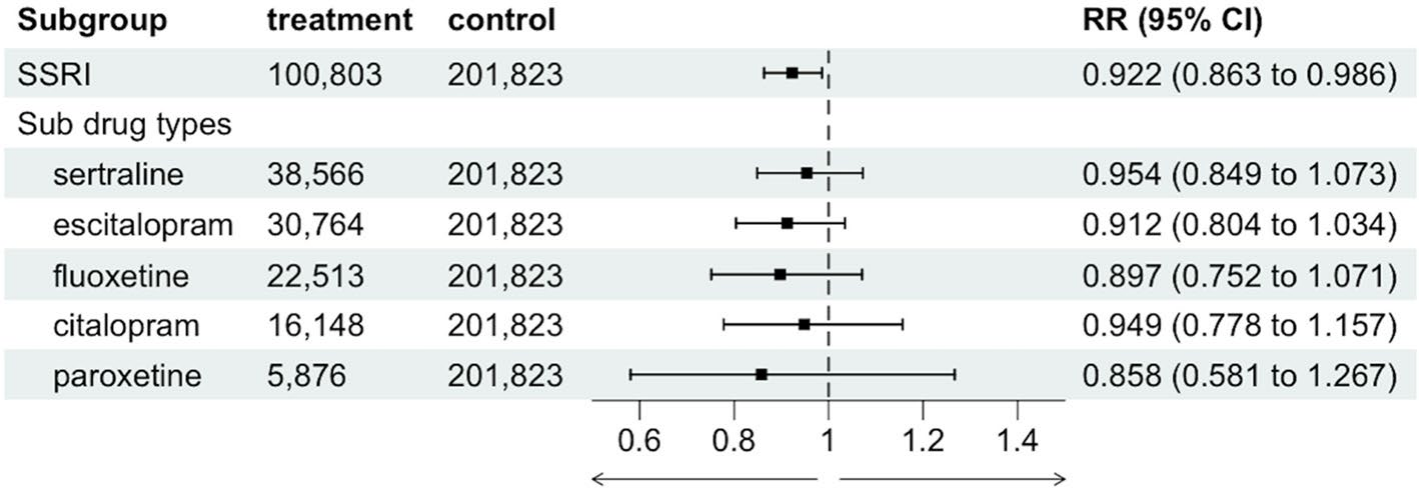
Relationship between SSRI use (overall and by SSRI type) and Long COVID among patients with depression

We found that SSRI users had a lower risk of Long COVID (adjusted risk ratio (aRR) 0.922, 95% confidence interval (CI) (0.863, 0.986)) compared to nonusers (Fig. 2). Adjustment for baseline confounders shifted the estimate a fair distance from the unadjusted association, which failed to detect relationship between SSRI use and Long COVID (unadjusted RR 1.042, 95% CI (0.984, 1.104)) (Table 2).

**Table 2:** Relationships between SSRI use during acute COVID-19 infection and 12-month long COVID risks

SSRI Type	Sample size Using Drug	Sample size not using drug	Unadjusted risk using drug	Unadjusted Risk not using Drug	Unadjusted RR	Unadjusted CI Lower	Unadjusted CI Upper	Adjusted risk using drug	Adjusted Risk not using drug	Adjusted RR	Adjusted CI Lower	Adjusted CI Upper
Any SSRI	100803	201823	0.0172	0.0165	1.0422	0.9840	1.1039	0.0169	0.0184	0.9225	0.8632	0.9858
New SSRI Prescription*	34646	201823	0.0166	0.0165	1.0104	0.9255	1.1030	0.0161	0.0181	0.88626	0.79783	0.9845
SSRI subgroups												
Fluoxetine	22513	201823	0.0172	0.0165	1.0409	0.9378	1.1554	0.0161	0.0180	0.8972	0.7516	1.0710
Sertraline	38566	201823	0.0162	0.0165	0.9798	0.9001	1.0665	0.0173	0.0181	0.9540	0.8486	1.0725
Paroxetine	5876	201823	0.0153	0.0165	0.9275	0.7535	1.1416	0.0155	0.0180	0.8578	0.5808	1.2671
Citalopram	16148	201823	0.0183	0.0165	1.1062	0.9831	1.2447	0.0172	0.0181	0.9486	0.7778	1.1570
Escitalopram	30764	201823	0.0177	0.0165	1.0747	0.9825	1.1755	0.0165	0.0181	0.9120	0.8041	1.0345

*SSRI* Selective serotonin reuptake inhibitor, *CI* Confidence interval *RR* Risk ratio

*New prescription for an SSRI 1-4 months before acute COVID-19 with no prior history of SSRI prescription

We evaluated the relationship between individual SSRI types and Long COVID (fluoxetine, sertraline, paroxetine, escitalopram, and citalopram). In our subgroup analysis, comparing the use of each of the five SSRIs to no SSRI use, we did not detect an association between the use of fluoxetine (aRR 0.897, 95% CI (0.752, 1.071)), sertraline (aRR 0.954, 95% CI (0.849, 1.073)), escitalopram (aRR 0.912, 95% CI (0.804, 1.034)), paroxetine (aRR 0.858, 95% CI (0.581, 1.267)), or citalopram (aRR 0.949, 95% CI (0.778, 1.157)) and the risk of Long COVID, although all point estimates indicated a protective (i.e., RR < 1), albeit not significant, relationship. We did not find evidence of a dose–response relationship between fluoxetine dose and risk of Long COVID (60 mg vs. 10 mg aRR 1.421, 95% CI (0.656, 3.080)) (see Additional file 1: Supplemental Table 2).

### Sensitivity analyses and confounding

We found that the relationship between SSRI use and Long COVID was strongly and qualitatively confounded, as the unadjusted estimate indicated a positive (harmful) correlation, but the adjusted estimate indicated a negative (protective) correlation. We observed the change in estimate following the backward exclusion of each covariate, where we defined “confounder RR” as the risk ratio in the fully adjusted model divided by the risk ratio of the partially adjusted model (with the covariate excluded) (see Additional file 1: Supplemental Table 3). We found that the strongest confounders of the relationship between SSRI use and Long COVID were baseline systemic corticosteroid use (confounder RR 0.983), monitoring during the outcome window (binary indicator of healthcare interactions between 1 and 12 months after acute COVID-19) (confounder RR 0.989), healthcare utilization at baseline (confounder RR 0.995), and social deprivation index (confounder RR 1.005). We also evaluated the impact of excluding two groups of covariates, healthcare utilization (number of healthcare interactions before baseline, healthcare interaction rate before baseline, and monitoring during the outcome window) and baseline general health and comorbidities general health and comorbidities (BMI, chronic lung disease, diabetes, obesity, immunocompromised status, smoking, corticosteroid use, hypertension, and COVID-19 vaccinations). We found that excluding factors related to healthcare utilization led to a confounder RR of 0.969 while excluding factors related to baseline comorbidities and health led to a confounder RR of 0.979.

In a subgroup analysis comparing new SSRI users (first SSRI prescription 1 to 4 months before acute COVID-19 with no prior history of SSRI use) to SSRI nonusers, we found a protective association similar to the primary analysis (aRR 0.886, 95% CI (0.780, 0.985)) (Table 2).

**Fig. 3:**
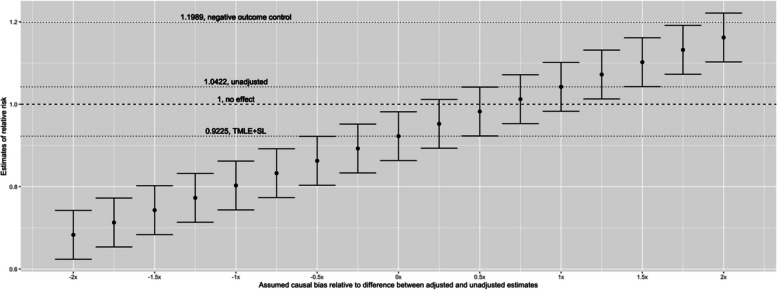
Nonparametric sensitivity analysis depicting the observed, adjusted risk ratio (TMLE + SL) as well as the unadjusted risk ratio (unadjusted) and the results of an analysis of a negative control outcome (bone fracture)

We conducted a nonparametric sensitivity analysis to evaluate the potential impact of bias on our results (Fig. 3). We found that 0.65 units of bias, where one unit corresponds to the difference between our adjusted and unadjusted estimate, could lead to a value as extreme as our observed estimate, due to random variation alone.

## Discussion

We found a protective effect of SSRI use at the time of acute SARS-CoV-2 infection on subsequent 12-month risk of Long COVID among patients with depression. These results are consistent with the hypothesis that SSRIs may be an effective intervention to prevent Long COVID, which also supports the hypothesis that serotonin may play a role in the development of Long COVID. Randomized controlled trials are currently underway to evaluate the ability of SSRIs to prevent or treat Long COVID (NCT05874037, NCT06128967). With ongoing COVID-19 transmission, the risk of Long COVID remains prevalent, and finding interventions to prevent Long COVID remains prudent. SSRIs may serve as an important tool in preventing this condition and limiting the rippling effects of the COVID-19 pandemic.

Our findings regarding the protective effect of SSRI use on Long COVID risk are consistent with previous studies. This observed treatment effect, a risk ratio of 0.922 (95% CI 0.863, 0.986), is less protective than a previous analysis, which found a risk ratio of 0.76 (95% CI 0.62, 0.90) [14]. The difference in these effects may be attributed to several potential factors, including the previous study’s use of predicted Long COVID status rather than observed diagnosis (yielding a prevalence of 15% rather than 2%) as well as our restriction to only include patients with a diagnosis of a depressive disorder [14]. These considerations may avoid bias and confounding due to indication, respectively.

The observed unadjusted and adjusted estimates varied. The unadjusted association indicating a non-significant relationship between SSRI use and Long COVID may be explained by strong confounding due to various factors and is supported by our finding of imbalance and confounding due to healthcare utilization, medication usage, and socioeconomic factors (see Additional file 1: Appendix 2 for details). Our finding that factors related to healthcare utilization rate (number of healthcare interactions before baseline and outcome monitoring indicator) were strong confounders of our observed relationship highlights the importance of addressing differential healthcare utilization rates and other causal considerations in observational studies that rely on Long COVID diagnosis as an outcome of interest [29]. We observed similar estimates in our overall analysis (aRR 0.92, 95% CI (0.86, 0.99)) and subgroup analysis comparing new SSRI users to SSRI nonusers (aRR 0.89, 95% CI (0.80, 0.98)). This finding supports that our observed associations were minimally biased by patient history of SSRI use, although the small difference in observed point estimates indicates that our observed association may be conservative (i.e., the true protective effect of SSRIs on Long COVID may be even stronger).

These findings provide support for the hypothesis that low serotonin may be a driver of Long COVID incidence and that SSRIs may prevent or treat Long COVID. This finding merits further exploration of the hypothesis of Wong et al. regarding Long COVID etiology via hypoactivity in the serotonin system [4]. This hypothesis posits that remnants of the SARS-CoV-2 virus leads to sustained release of viral RNA-induced type 1 interferons, which decreases tryptophan uptake and prevents cortisol production. According to this hypothesis, SSRI use may interrupt this causal pathway of disease etiology [4]. As this hypothesis posits that low serotonin is a downstream effect of lingering SARS-CoV-2 virus and sustained interferon 1 response, these findings also hint at interventions that aim to detect or treat persistent viral load of SARS-CoV-2 or viral RNA-induced type 1 interferon.

These findings indicate the need for several future studies to further explore these hypotheses. The impact of SSRIs on Long COVID risk in other populations, such as premenstrual dysphoric disorder or generalized anxiety disorder, may provide additional insights regarding the generalizability of these findings. In addition, future investigations should evaluate the impact of other serotonergic drugs, such as serotonin-norepinephrine reuptake inhibitors (SNRIs), on Long COVID risk. Finally, given the phenotypic overlap between Long COVID and other post-infectious chronic somatoform disorders, such as chronic Lyme’s disease (patients with both conditions frequently exhibit post-exertional malaise, chronic pain, brain fog, etc.), investigators should investigate the ability of SSRIs to treat or prevent these related conditions [34–36]. It should be noted that investigators have found mixed results regarding long-term effects of SSRIs on the serotonin system, with some studies indicating that long-term SSRI use may lead to decreased serotonin signaling (i.e., a negative feedback loop) [37–41].

We did not find evidence of heterogeneity of the relationship between SSRI use and Long COVID depending on SSRI type, which is consistent with previous findings [14]. A previous study did not find a differential impact of moderate to high-affinity sigma 1 receptor agonist SSRIs (fluvoxamine, fluoxetine, escitalopram, and citalopram) versus non-high affinity SSRIs (sertraline and paroxetine) in their relationship with Long COVID [14]. We caution readers to consider this finding in the context of a few limitations. Residual confounding due to indication, as different depressive symptomatology, comorbidities, side effects, and tolerance may lead providers to prescribe a specific SSRI over another SSRI. For instance, citalopram and paroxetine are often prescribed for obsessive–compulsive disorder, which may be associated with Long COVID symptoms [42–45]. Furthermore, our analysis of paroxetine was limited by a small sample size of users (*n* = 7189). We also did not find evidence of a dose–response relationship between fluoxetine and Long COVID. This may be explained due to a large proportion of missingness of dose information leading to a small functional sample size. Future studies should further explore the possibility of a dose–response relationship [9].

### Strengths and limitations

A strength of this study is its large, national sample size of patients and the broad range of high-dimensional data that we included via N3C. This rich data source allows us to construct a cohort of patients with a diagnosis of a depressive condition, assess their SSRI use at the time of SARS-CoV-2 infection, and evaluate their probability of Long COVID diagnosis. Furthermore, the documentation of comorbidities, sociodemographic information, and other medical history allows for rigorous multivariate adjustment.

A second strength of this study is the analytic methods that we applied. A Targeted Learning approach, involving Super Learner and targeted maximum likelihood estimation, allows for efficient and flexible estimation while making minimal parametric assumptions [19, 20, 23, 31, 46]. With this large sample size of high-dimensional data, this allows us to aggressively reduce bias due to measured, potentially high-dimensional confounding and to do so with nearly no model assumptions. These methods allowed us to intervene on participant observation during the outcome window, which is an important driver of differential outcome ascertainment [29, 33]. Furthermore, nonparametric sensitivity analyses allowed us to determine the extent to which our results are vulnerable to bias. Cumulatively, these methods provide a replicable framework for investigators to conduct rigorous observational analyses using electronic health record databases such as N3C.

A third strength of this study was its ability to flexibly account for and intervene on the missingness of the outcome and heterogeneous monitoring [33]. There is significant heterogeneity in N3C’s documentation of Long COVID diagnoses (our outcome of interest), as is common with electronic health record databases. Previous studies have found that Long COVID diagnosis is strongly correlated with healthcare utilization rate [29, 47]. We sought to control for healthcare utilization rate by adjusting for multiple factors related to healthcare utilization, including healthcare visits per month before SARS-CoV-2 infection. In addition, we were able to use novel causal inference framing to define our parameter of interest at the ratio of probabilities of PASC under universal monitoring, i.e., by “intervening” on whether an individual had a healthcare visit between 1 and 12 months following acute SARS-CoV-2 infection (the period at-risk for Long COVID), to observe the counterfactual impact of SSRI exposure given that all patients were observed during the period at-risk for the outcome [48]. It remains possible that residual confounding due to healthcare utilization rate remains, although this would likely bias our estimate towards the null, indicating that our observed measure of association is likely conservative [29].

This study had several limitations. We defined the exposure of interest as a binary, time-invariant variable based on SSRI use during COVID-19. It remains possible that factors related to the duration of SSRI use, timing of SSRI use, or SSRI dosage may modify this relationship, although these factors are poorly documented (i.e., high missingness) in EHR databases such as N3C and should be explored in a future study. Furthermore, PASC diagnosis (ICD code U09.9) has limited sensitivity and low clinical utilization, which may lead to outcome misclassification. Furthermore, the binary definition of Long COVID may fail to reflect heterogeneity within Long COVID subtypes (e.g., neurological versus gastrointestinal symptoms). Finally, as an observational study, this analysis may be subject to residual bias and investigators should conduct randomized controlled trials to corroborate these findings. The generalizability of N3C patients has been described previously [6, 47, 49]. N3C is a broad, national sample of patients, as it relies on electronic health record data, but it skews towards patients who engage more with healthcare systems. This yields a study population that is generally older, has more comorbidities than the general population, and underrepresents un- or underinsured patients [47].

## Conclusions

This study suggests that the use of SSRIs during acute COVID-19 is associated with a lower risk of Long COVID among patients with depression. These results support the hypothesis that serotonin may be a mechanistic biomarker of Long COVID and that SSRIs may be an effective intervention to prevent Long COVID.

Supplementary information.

Additional file 1: Supplemental Table 1 List of depressive conditions. Appendix 1 Covariate information. Supplemental Fig. 1 CONSORT diagram. Supplemental Table 2 Exploratory analysis of dose–response relationship between fluoxetine use and Long COVID. Supplemental Table 3 Variable importance of included covariates. Appendix 2 Nonparametric sensitivity analysis.

## Supplementary Information

Additional file 1: Supplemental Table 1 – List of depressive conditions, Appendix 1 – Covariate information, Supplemental Figure 1 – CONSORT diagram, Supplemental Table 2 - Exploratory analysis of dose-response relationship between fluoxetine use and Long COVID, Supplemental Table 3 - Variable importance of included covariates, Appendix 2 – Nonparametric sensitivity analysis.

## Data Availability

All analytic code and data are available in the N3C Enclave by request. Access to the N3C Data Enclave is managed by NCATS (https://ncats.nih.gov/research/research-activities/n3c/resources/data-access). Interested researchers must first complete a data use agreement, and next a data use request, in order to access the N3C Data Enclave. Once access is granted, the N3C data use committee must review and approve all use of data and the publication committee must approve all publications involving N3C data.

## Abbreviations

SSRI: Selective serotonin reuptake inhibitor
COVID-19: Coronavirus disease 2019
PASC: Post-acute sequelae of COVID-19
EHR: Electronic health record
RR: Risk ratio
