# Supplementary material for: SSRI use during acute COVID-19 and risk of Long COVID among patients with depression
Source: BMC Med. 2024 Oct 8;22:445. doi: 10.1186/s12916-024-03655-x (PMC11462648; doi:10.1186/s12916-024-03655-x)
Supplement: Supplementary file 1 — Additional file 1: Supplemental Table 1 – List of depressive conditions, Appendix 1 – Covariate information, Supplemental Figure 1 – CONSORT diagram, Supplemental Table 2 - Exploratory analysis of dose-response relationship between fluoxetine use and Long COVID, Supplemental Table 3 - Variable importance of included covariates, Appendix 2 – Nonparametric sensitivity analysis. [file 12916_2024_3655_MOESM1_ESM.pdf]

Supplemental Table 1. List of depressive conditions

Concept Name	Concept Code	Concept Id	Domain Id	Vocabulary Id	Concept Class Id
Recurrent major depressive disorder co-occurrent with anxi	16265061000119105	35615154	Condition	SNOMED	Disorder
Major depression, melancholic type	320751009	4154391	Condition	SNOMED	Disorder
Severe major depression, single episode, without psychotic	76441001	441534	Condition	SNOMED	Disorder
Secondary dysthymia late onset	36170009	4263770	Condition	SNOMED	Disorder
Recurrent major depressive episodes, severe, with psychos	191613003	434911	Condition	SNOMED	Disorder
Reactive depression, first episode	1086691000000102	35609845	Condition	SNOMED	Disorder
Recurrent major depressive episodes, mild	191610000	438998	Condition	SNOMED	Disorder
Reactive depression (situational)	87414006	4314692	Condition	SNOMED	Disorder
Depressive disorder in mother complicating childbirth	10835871000119104	45757213	Condition	SNOMED	Disorder
Depressive disorder caused by drug	191495003	4103126	Condition	SNOMED	Disorder
Mood disorder with depressive symptoms caused by volatil	762339009	42538599	Condition	SNOMED	Disorder
Persistent depressive disorder	1153575004	607543	Condition	SNOMED	Disorder
Recurrent depression with current severe episode and psy	1089511000000100	35610097	Condition	SNOMED	Disorder
Major depression in partial remission	30605009	4148630	Condition	SNOMED	Disorder
Severe major depression without psychotic features	75084000	4327337	Condition	SNOMED	Disorder
Recurrent major depressive episodes, severe	764611000000100	44805542	Condition	SNOMED	Disorder
Major depression with psychotic features	726772006	37111697	Condition	SNOMED	Disorder
Reactive depression, prolonged single episode	1086661000000108	35609842	Condition	SNOMED	Disorder
Endogenous depression	300706003	4114950	Condition	SNOMED	Disorder
Depressive conduct disorder	231542000	4333687	Condition	SNOMED	Disorder
Severe seasonal affective disorder	133121000119109	44782720	Condition	SNOMED	Disorder
Depressive disorder caused by amphetamine	16238181000119101	37209503	Condition	SNOMED	Disorder
Major depression in full remission	63412003	4269493	Condition	SNOMED	Disorder
Secondary dysthymia	85080004	4224639	Condition	SNOMED	Disorder
Agitated depression	83458005	4308866	Condition	SNOMED	Disorder
Generalized neuromuscular exhaustion syndrome	87842000	4336980	Condition	SNOMED	Disorder
Mild recurrent major depression	40379007	4228802	Condition	SNOMED	Disorder
Reactive depressive psychosis, single episode	288751000119101	43020483	Condition	SNOMED	Disorder
Severe major depression, single episode	251000119105	42872411	Condition	SNOMED	Disorder
Atypical depressive disorder	191659001	438727	Condition	SNOMED	Disorder
Severe major depression with psychotic features, mood-inc	60099002	4243822	Condition	SNOMED	Disorder
Exacerbation of depressive disorder	OMOP5165960	1340305	Condition	OMOP Extension	Disorder
Recurrent brief depressive disorder	40568001	4226155	Condition	SNOMED	Disorder
Mood disorder with depressive symptoms caused by halluc	762336002	42538596	Condition	SNOMED	Disorder
Recurrent major depression in remission	68019004	433991	Condition	SNOMED	Disorder
Recurrent major depressive disorder with postpartum onset	71336009	4324959	Condition	SNOMED	Disorder
Late onset dysthymia	19694002	4057218	Condition	SNOMED	Disorder
Major depression single episode, in partial remission	70747007	4323418	Condition	SNOMED	Disorder
Multi-infarct dementia with depression	14070001	443864	Condition	SNOMED	Disorder
Severe recurrent major depression without psychotic featur	36474008	435220	Condition	SNOMED	Disorder
Recurrent moderate major depressive disorder co-occurren	16264901000119109	35615153	Condition	SNOMED	Disorder
Severe major depression, single episode, with psychotic fe	77911002	4299785	Condition	SNOMED	Disorder
Primary dysthymia late onset	67711008	4195680	Condition	SNOMED	Disorder
Reactive depression, recurrent	1086681000000104	35609844	Condition	SNOMED	Disorder
Chronic recurrent major depressive disorder	2618002	4094358	Condition	SNOMED	Disorder
Moderate major depression, single episode	15639000	4049623	Condition	SNOMED	Disorder
Mood disorder with depressive symptoms caused by cocair	724690002	37110438	Condition	SNOMED	Disorder
Recurrent depression with current severe episode without p	1089631000000109	35610108	Condition	SNOMED	Disorder
Severe recurrent major depression	281000119103	43531624	Condition	SNOMED	Disorder
Moderate depression	310496002	4151170	Condition	SNOMED	Disorder
Dysthymia	78667006	433440	Condition	SNOMED	Disorder
Major depressive disorder, single episode with postpartum	25922000	4093584	Condition	SNOMED	Disorder
Recurrent reactive depressive episodes, severe, with psych	1086471000000103	35609824	Condition	SNOMED	Disorder
Perinatal depression	10211000132109	36712668	Condition	SNOMED	Disorder
Recurrent mild major depressive disorder co-occurrent with	16264621000119109	35615151	Condition	SNOMED	Disorder
Recurrent major depressive disorder in partial remission co-	16265301000119106	35615155	Condition	SNOMED	Disorder
Recurrent major depression	66344007	4282316	Condition	SNOMED	Disorder
Chronic depressive personality disorder	442057004	40481798	Condition	SNOMED	Disorder
Recurrent depression with current moderate episode	1089641000000100	35610109	Condition	SNOMED	Disorder
Postoperative depression	82218004	4305966	Condition	SNOMED	Disorder
Reactive depression, single episode	1086671000000101	35609843	Condition	SNOMED	Disorder
Seasonal affective disorder	247803002	4092239	Condition	SNOMED	Disorder
Menopausal depression	84788008	4223090	Condition	SNOMED	Disorder
Severe recurrent major depression with psychotic features,	15193003	4034842	Condition	SNOMED	Disorder
Recurrent major depressive episodes	268621008	432285	Condition	SNOMED	Disorder
Severe major depression, single episode, with psychotic fe	20250007	4067409	Condition	SNOMED	Disorder
Moderate recurrent major depression	18818009	4077577	Condition	SNOMED	Disorder
Minimal major depression	720455008	36715000	Condition	SNOMED	Disorder

Minimal major depression single episode	720454007	36714999	Condition	SNOMED	Disorder
Mood disorder with major depressive-like episode due to ge	77486005	4298317	Condition	SNOMED	Disorder
Major depression in remission	42810003	4176002	Condition	SNOMED	Disorder
Antenatal depression	788120007	37312479	Condition	SNOMED	Disorder
Recurrent major depressive disorder with atypical features	38694004	4304140	Condition	SNOMED	Disorder
Endogenous depression first episode	231499006	4333679	Condition	SNOMED	Disorder
Mood disorder with depressive symptoms caused by amphe	838530009	3654788	Condition	SNOMED	Disorder
Depressive disorder in mother complicating pregnancy	94631000119100	37018656	Condition	SNOMED	Disorder
Moderately severe recurrent major depression	720452006	36714998	Condition	SNOMED	Disorder
Recurrent major depression in partial remission	33135002	4141454	Condition	SNOMED	Disorder
Stuporous depression	79842004	4197222	Condition	SNOMED	Disorder
Depressive disorder	35489007	440383	Condition	SNOMED	Disorder
Single major depressive episode, in remission	764711000000106	44805550	Condition	SNOMED	Disorder
Recurrent severe major depressive disorder co-occurrent w	16264821000119108	35615152	Condition	SNOMED	Disorder
Severe major depressive disorder co-occurrent with anxiety	16266991000119108	37109054	Condition	SNOMED	Disorder
Recurrent major depressive disorder with catatonic features	39809009	4220023	Condition	SNOMED	Disorder
Recurrent major depression in full remission	46244001	4263748	Condition	SNOMED	Disorder
Recurrent major depressive episodes, in remission	764701000000109	44813499	Condition	SNOMED	Disorder
Moderate major depression	832007	4307111	Condition	SNOMED	Disorder
Minimal depression	718636001	36713698	Condition	SNOMED	Disorder
Mixed anxiety and depressive disorder	231504006	4338031	Condition	SNOMED	Disorder
Mild major depression, single episode	79298009	4195572	Condition	SNOMED	Disorder
Severe postnatal depression	237350002	4129184	Condition	SNOMED	Disorder
Mood disorder with depressive symptoms caused by sedati	724676000	37117211	Condition	SNOMED	Disorder
Major depressive disorder, single episode with atypical feat	42925002	4181807	Condition	SNOMED	Disorder
Chronic depression	192080009	4103574	Condition	SNOMED	Disorder
Mood disorder with depressive symptoms caused by stimula	762329003	42538590	Condition	SNOMED	Disorder
Mild postnatal depression	237349002	4129842	Condition	SNOMED	Disorder
Mood disorder with depressive symptoms caused by synthe	762512002	42538736	Condition	SNOMED	Disorder
Reactive depressive psychosis	191676002	435520	Condition	SNOMED	Disorder
Premenstrual dysphoric disorder in remission	426578000	4145216	Condition	SNOMED	Disorder
Early onset dysthymia	2506003	4096229	Condition	SNOMED	Disorder
Recurrent major depressive episodes, severe, with psychos	755331000000108	44805669	Condition	SNOMED	Disorder
Mild depression	310495003	4149320	Condition	SNOMED	Disorder
Severe major depression, single episode, with psychotic fe	430852001	438406	Condition	SNOMED	Disorder
Single major depressive episode, severe, with psychosis, p	755321000000106	44805668	Condition	SNOMED	Disorder
Single episode of major depression in full remission	19527009	4025677	Condition	SNOMED	Disorder
Major depressive disorder in mother complicating childbirth	10811121000119102	45757195	Condition	SNOMED	Disorder
Mood disorder with depressive symptoms caused by hypno	724677009	37110428	Condition	SNOMED	Disorder
Mild major depressive disorder co-occurrent with anxiety sin	16265951000119109	37109052	Condition	SNOMED	Disorder
Single major depressive episode, severe, with psychosis	191604000	439259	Condition	SNOMED	Disorder
Mood disorder with mixed manic and depressive symptoms	723930006	37109952	Condition	SNOMED	Disorder
Primary dysthymia	83176005	4307951	Condition	SNOMED	Disorder
Major depressive disorder	370143000	4152280	Condition	SNOMED	Disorder
Mood disorder with depressive symptoms caused by dissoc	762345001	42538604	Condition	SNOMED	Disorder
Post-schizophrenic depression	231485007	4332994	Condition	SNOMED	Disorder
Postpartum major depression in remission	104851000119103	42534817	Condition	SNOMED	Disorder
Moderately severe major depression	719592004	36714389	Condition	SNOMED	Disorder
Depressive disorder caused by methamphetamine	16238221000119109	37309680	Condition	SNOMED	Disorder
Maternity blues	279225001	4133073	Condition	SNOMED	Disorder
Secondary dysthymia early onset	3109008	4150047	Condition	SNOMED	Disorder
Treatment resistant depression	1153570009	607540	Condition	SNOMED	Disorder
Major depressive disorder in mother complicating pregnanc	10811161000119107	45757196	Condition	SNOMED	Disorder
Moderately severe major depression single episode	720453001	36717389	Condition	SNOMED	Disorder
Recurrent depression	191616006	4098302	Condition	SNOMED	Disorder
Exacerbation of major depressive disorder	OMOP5166047	1340392	Condition	OMOP Extension	Disorder
Major depressive disorder, single episode with melancholic	63778009	4270907	Condition	SNOMED	Disorder
Severe depression	310497006	4149321	Condition	SNOMED	Disorder
Mood disorder with depressive symptoms caused by alcoho	723928009	37109950	Condition	SNOMED	Disorder
Recurrent major depressive episodes, in partial remission	764691000000109	44805549	Condition	SNOMED	Disorder
Severe major depression with psychotic features	73867007	4250023	Condition	SNOMED	Disorder
Acute depression	712823008	37016718	Condition	SNOMED	Disorder
Moderate major depressive disorder co-occurrent with anxie	16266831000119100	37109053	Condition	SNOMED	Disorder
Postviral depression	192079006	4102973	Condition	SNOMED	Disorder
Depressive disorder in remission	698957003	44782943	Condition	SNOMED	Disorder
Endogenous depression - recurrent	274948002	4168858	Condition	SNOMED	Disorder
Minimal recurrent major depression	720451004	36714997	Condition	SNOMED	Disorder
Severe major depression	450714000	42872722	Condition	SNOMED	Disorder
Recurrent major depressive disorder with melancholic featu	319768000	4205471	Condition	SNOMED	Disorder
Chronic major depressive disorder, single episode	14183003	4031328	Condition	SNOMED	Disorder

Postpartum depression	58703003	4239471	Condition	SNOMED	Disorder
Involutional depression	321717001	4154805	Condition	SNOMED	Disorder
Major depression, single episode	36923009	4282096	Condition	SNOMED	Disorder
Primary dysthymia early onset	38451003	4243308	Condition	SNOMED	Disorder
Severe recurrent major depression with psychotic features	28475009	4154309	Condition	SNOMED	Disorder
Severe recurrent major depression with psychotic features,	33078009	4141292	Condition	SNOMED	Disorder
Minor depressive disorder	48589009	4174987	Condition	SNOMED	Disorder
Severe major depression with psychotic features, mood-co	33736005	4144233	Condition	SNOMED	Disorder
Mood disorder with depressive features due to general me	75837004	4328217	Condition	SNOMED	Disorder
Mood disorder with depressive symptoms caused by anxiol	724678004	37110429	Condition	SNOMED	Disorder
Mood disorder with depressive symptoms caused by opioid	762321000	42538584	Condition	SNOMED	Disorder
Recurrent major depressive episodes, moderate	191611001	432883	Condition	SNOMED	Disorder
Moderately severe depression	719593009	36717092	Condition	SNOMED	Disorder
Premenstrual dysphoric disorder	596004	4242733	Condition	SNOMED	Disorder
Major depressive disorder, single episode with catatonic fe	69392006	4287238	Condition	SNOMED	Disorder
Masked depression	231500002	4338029	Condition	SNOMED	Disorder
Mild major depression	87512008	4336957	Condition	SNOMED	Disorder

Appendix 1. Covariate information

Unless otherwise stated, all variables were evaluated before baseline (index acute SARS-CoV-2 infection).

Selective serotonin reuptake inhibitor (SSRI) use (code sets 602742506, 709408353, 402078260, 738475452, 255742597, 408043043, 444624587): We defined SSRI users as individuals who were using an SSRI medication (fluoxetine, sertraline, paroxetine, citalopram, escitalopram, fluvoxamine, and vilazodone, phenotyped using RxNorm) at the time of SARS-CoV-2 infection (beginning at least 30 days prior to SARS-CoV-2 infection) and we defined all other individuals as nonusers.

Long COVID Diagnosis (post-acute sequelae of COVID-19, PASC, ICD-10 code U09.9): We defined Long COVID diagnosis as between 1 and 12 months following acute SARS-CoV-2 infection.

Number of visits pre-SARS-CoV-2 infection: The number of healthcare visits that a participant attended between the start of N3C monitoring (January 1, 2018) and incident SARS-CoV-2 infection. We define a healthcare visit as an interaction (or cluster of interactions) with a healthcare provider that was associated with a given medical condition, diagnosis, or procedure.

Healthcare visits per month before SARS-CoV-2 infection): The number of healthcare visits that a participant attended between the start of N3C monitoring (January 1, 2018) and incident SARS-CoV-2 infection divided by the number of months between their first medical visit documented in N3C and their incident SARS-CoV-2 infection.

Sex: Participant biologic sex.

Age at acute SARS-CoV-2 infection: Patient age in years.

Race/ ethnicity: A covariate that combines a patient’s categorical race status and categorical ethnicity.

Common data model format: The common data model documentation format used by the contributing data provider.

Region of residence: A categorical measure of geographic region, denoted by the first digits of the patient zip code.

Body mass index (BMI): A continuous measure of body composition.

Tobacco smoking status: A binary indicator denoting current tobacco smoking status (user vs. nonuser)

Obesity: A binary indicator denoting a previous diagnosis of obesity.

Diabetes: A binary indicator denoting a previous diagnosis of diabetes.

Chronic lung disease: A binary indicator denoting a previous diagnosis of chronic lung disease.

Heart failure: A binary indicator denoting a previous diagnosis with heart failure.

Hypertension: A binary indicator of hypertension diagnosis.

Use of systemic corticosteroids: A binary indicator of whether a patient was currently receiving systemic corticosteroids.

Whether the patient was immunocompromised: A binary indicator variable denoting whether the patient was diagnosed with having a comprised immune status.

Number of COVID-19 vaccination doses before infection: The number of COVID-19 vaccination and booster doses that the patient received.

Percent of the county with an income level below the poverty line: County-level variable indicating what percent of the county received an income below the federal poverty line.

Social deprivation index score: County-level variable that quantifies social deprivation and inequality.

Depression severity score (code sets 649312011, 924814934, 937942059): A three-level categorical variable denoting the patient’s severity of major depressive disorder. Levels include “mild,” “moderate,” and “severe.” Depression severity was evaluated prior to SSRI use.

Anxiety (code set 964465007): An indicator of whether a patient was diagnosed with an anxiety disorder prior to SSRI prescription.

Antipsychotic medication use (code set 241164369): An indicator variable denoting whether a patient had a history of antipsychotic medication use prior to SSRI prescription.

Benzodiazepine medication use (code set 778556406): An indicator variable denoting whether a patient had a history of benzodiazepine medication use prior to SSRI prescription.

Post-COVID visit indicator: An indicator variable denoting whether the patient had a documented healthcare visit between 1 and 12 months following index acute SARS-CoV-2 infection (the outcome observation period).

Bone fracture (code set 889591431): A binary variable indicating a bone fracture between 1 and 12 months following SARS-CoV-2 infection.

CONSORT Diagram

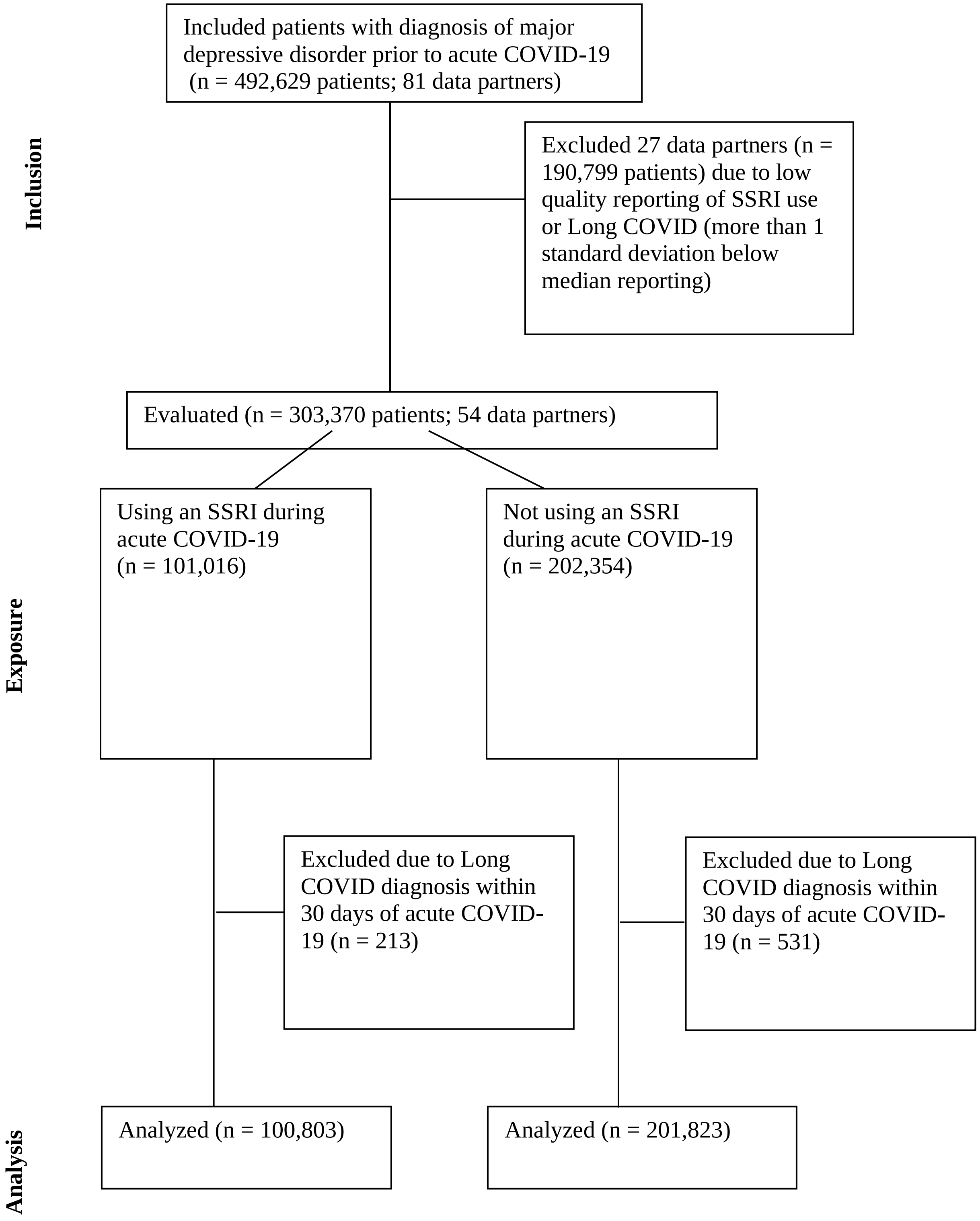

Supplemental Table 2. Exploratory analysis of dose-response relationship between fluoxetine use and Long COVID.

Drug	Dose	N (RR v.s no SSRI use)	N (RR v.s 10 mg dosage)	RR Vs. No SSRI Use (95% CI)	RR Vs. 10 mg dose (95% CI)	RD vs. no SSRI Use (95% CI)	RD vs. 10 mg dose (95% CI)
Fluoxetine	10	568682	NA	0.84916 (0.65182, 1.1063)	NA	-0.0032206 (-0.00802, 0.0015789)	NA
	20	579502	19952	0.88769 (0.7836, 1.0056)	0.93935 (0.75283, 1.1721)	-0.0024068 (-0.0047859, -2.7705e-05)	-0.001528 (-0.0070363, 0.0039802)
	40	572562	13012	0.90261 (0.72046, 1.1308)	1.0164 (0.80321, 1.2861)	-0.0020847 (-0.0064427, 0.0022732)	0.00042721 (-0.0057503, 0.0066048)
	60	564526	4976	0 (NA)	1.4207 (0.65529, 3.0803)	-0.021348 (-0.032384, -0.010313)	0.0099832 (-0.015715, 0.035682)

Supplemental Table 3. Variable importance of included covariates

Feature	psi	lower	upper	p_value	psi_ratio	psi_diff	t_statistics	p_value_diff	absolute difference
FULL	0.90101871	0.86065944	0.94327057	8.28E-06	1	0	1	FULL	
SYSTEMICCORTICOSTEROIDS_before_or_day_of_covid_indicator	0.91624483	0.87533266	0.9590692	0.0001746	0.98338204	-0.0152261	-369.7215942	0	0.015226123
post_COVID_visit_indicator	0.91122853	0.87040224	0.95396977	7.04E-05	0.98879555	-0.0102098	-248.1792109	0	0.010209814
number_of_visits_before_covid	0.90560244	0.86506316	0.94804151	2.20E-05	0.99493847	-0.0045837	-111.8177355	0	0.00458373
sdi_score	0.89670375	0.85679962	0.93846636	2.67E-06	1.00481202	0.00431496	106.0927592	0	0.00431496
month_number_10	0.89754184	0.85732695	0.9396431	3.80E-06	1.00387377	0.00347687	85.1580336	0	0.003476871
month_number_8	0.89768978	0.85745514	0.93981235	3.96E-06	1.00370834	0.00332894	81.51474815	0	0.003328936
month_number_9	0.89786198	0.85762451	0.93998729	4.11E-06	1.00351583	0.00315673	77.29526585	0	0.00315673
poverty_rate	0.89806277	0.85798557	0.94001201	3.91E-06	1.00329147	0.00295594	72.52267035	0	0.002955942
month_number_12	0.8983808	0.85811305	0.94053815	4.65E-06	1.00293629	0.00263791	64.56731375	0	0.002637911
month_number_11	0.89839397	0.85811954	0.9405586	4.68E-06	1.0029216	0.00262475	64.23974794	0	0.002624745
month_number_13	0.89859953	0.85831312	0.94077684	4.91E-06	1.00269217	0.00241919	59.19996962	0	0.002419186
month_number_4	0.89876888	0.85848788	0.9409399	5.06E-06	1.00250324	0.00224983	55.05934933	0	0.00224983
OBESITY_before_or_day_of_covid_indicator	0.90243912	0.86202594	0.94474694	1.13E-05	0.99842604	-0.0014204	-34.70427584	9.71E-264	0.001420407
OTHERIMMUNOCOMPROMISED_before_or_day_of_covid_indicator	0.9023589	0.86196513	0.94464563	1.10E-05	0.99851479	-0.0013402	-32.75222571	3.75E-235	0.00134019
month_number_3	0.89974231	0.8594204	0.94195603	6.30E-06	1.00141863	0.0012764	31.22107949	6.97E-214	0.0012764
CHRONICLUNGDISEASE_before_or_day_of_covid_indicator	0.89991176	0.85958835	0.94212675	6.52E-06	1.00123007	0.00110695	27.07584386	2.17E-161	0.001106952
TOBACCOSMOKER_before_or_day_of_covid_indicator	0.89993266	0.85958209	0.94217737	6.64E-06	1.00120682	0.00108606	26.55577632	2.49E-155	0.001086055
month_number_7	0.90000561	0.85965266	0.94225276	6.75E-06	1.00112567	0.00101311	24.77131173	2.01E-135	0.001013106
month_number_14	0.90009201	0.85975349	0.94232316	6.81E-06	1.00102957	0.0009267	22.66277784	1.11E-113	0.000926705
month_number_6	0.9001049	0.85976675	0.94233562	6.83E-06	1.00101523	0.00091381	22.34760026	1.36E-110	0.000913812
month_number_5	0.90014998	0.85983	0.94236068	6.82E-06	1.00096509	0.00086873	21.24986853	3.48E-100	0.00086873
BMI_max_observed_or_calculated_before_or_day_of_covid	0.90034418	0.8600001	0.94258088	7.18E-06	1.00074919	0.00067453	16.494632	4.08E-61	0.000674529
age_at_covid	0.90039427	0.86008305	0.94259484	7.13E-06	1.00069352	0.00062444	15.27599425	1.12E-52	0.00062444
HEARTFAILURE_before_or_day_of_covid_indicator	0.90045936	0.86013211	0.94267735	7.29E-06	1.00062119	0.00055935	13.68103658	1.33E-42	0.000559354
visits_per_month	0.90154733	0.86117	0.94381781	9.28E-06	0.99941366	-0.0005286	-12.92115605	3.44E-38	0.000528614
number_of_COVID_vaccine_doses_before_or_day_of_covid	0.9014545	0.86119243	0.94359887	8.57E-06	0.99951658	-0.0004358	-10.6673334	1.45E-26	0.000435785
month_number_1	0.90144469	0.86105896	0.9437246	9.13E-06	0.99952745	-0.000426	-10.41126221	2.21E-25	0.000425976
region_1	0.9006689	0.86031566	0.94291491	7.70E-06	1.00038839	0.00034981	8.553242783	1.20E-17	0.000349815
region_3	0.90074599	0.86038926	0.94299567	7.83E-06	1.00030277	0.00027272	6.667924443	2.60E-11	0.00027272
region_0	0.90075838	0.86040135	0.94300835	7.85E-06	1.00028902	0.00026034	6.365109871	1.95E-10	0.000260336
race_ethnicity_American_Indian_or_Alaska_Native_Non_Hispanic	0.90076423	0.86040681	0.94301461	7.87E-06	1.00028252	0.00025449	6.222049438	4.91E-10	0.000254486
region_5	0.9007757	0.86041843	0.9430259	7.88E-06	1.00026978	0.00024301	5.941499071	2.83E-09	0.000243011
cdm_name_TRINETX	0.90125414	0.86088411	0.94351728	8.72E-06	0.99973877	-0.0002354	-5.7552772	8.65E-09	0.000235431
race_ethnicity_Asian_Non_Hispanic	0.9012369	0.8608672	0.94349971	8.69E-06	0.9997579	-0.0002182	-5.333841201	9.62E-08	0.000218191
region_N	0.9008184	0.86045794	0.943072	7.96E-06	1.00022237	0.00020031	4.897322431	9.72E-07	0.000200311
depression_severity_Severe	0.90082749	0.86047065	0.94307711	7.96E-06	1.00021227	0.00019122	4.675219206	2.94E-06	0.000191218
race_ethnicity_White_Non_Hispanic	0.90082992	0.86046886	0.94308416	7.98E-06	1.00020957	0.00018879	4.615644152	3.92E-06	0.000188791
region_2	0.90083058	0.86046996	0.94308432	7.98E-06	1.00020884	0.00018813	4.599597378	4.23E-06	0.000188134
sex_OTHER	0.90084141	0.86048165	0.94309419	8.00E-06	1.00019682	0.00017731	4.334905349	1.46E-05	0.000177305
region_4	0.9011913	0.86082275	0.94345294	8.61E-06	0.99980849	-0.0001726	-4.219057976	2.45E-05	0.000172586
cdm_name_PCORNET	0.90085346	0.86049703	0.94310257	8.00E-06	1.00018343	0.00016525	4.040277915	5.34E-05	0.000165248
sex_UNKNOWN	0.90085358	0.86049704	0.94310282	8.00E-06	1.0001833	0.00016513	4.037324393	5.41E-05	0.000165127
race_ethnicity_Hispanic_or_Latino_Any_Race	0.90116243	0.86079645	0.94342131	8.55E-06	0.99984052	-0.0001437	-3.513403951	0.000442424	0.000143716
HYPERTENSION_before_or_day_of_covid_indicator	0.90115695	0.86078773	0.9434194	8.55E-06	0.9998466	-0.0001382	-3.379374302	0.000726538	0.000138239
month_number_2	0.90088128	0.86052425	0.94313098	8.05E-06	1.00015255	0.00013743	3.360123463	0.000779105	0.00013743
region_7	0.90088786	0.86052834	0.94314028	8.07E-06	1.00014525	0.00013085	3.1991287	0.001378479	0.00013085

cdm_name_OMOP	0.90089106	0.86053496	0.94313972	8.06E-06	1.0001417	0.00012765	3.12108355	0.00180192	0.000127652
depression_severity_Moderately	0.90113192	0.8607647	0.94339225	8.50E-06	0.99987437	-0.0001132	-2.767637746	0.00564652	0.000113212
region_9	0.90090854	0.86054547	0.94316481	8.12E-06	1.00012229	0.00011017	2.693421277	0.007072401	0.00011017
depression_severity_Missing	0.90090946	0.86055129	0.94316036	8.10E-06	1.00012126	0.00010925	2.671018546	0.007562268	0.000109247
sex_No_matching_concept	0.90112708	0.86076388	0.94338301	8.48E-06	0.99987974	-0.0001084	-2.649412861	0.008063301	0.00010837
race_ethnicity_Other_Non_Hispanic	0.90092766	0.86056452	0.94318394	8.15E-06	1.00010107	9.11E-05	2.22610905	0.026007097	9.10557E-05
bd_indicator	0.90094431	0.86060434	0.94317518	8.08E-06	1.00008258	7.44E-05	1.819371669	0.068855041	7.43973E-05
cdm_name_OMOP_PEDSNET	0.90108913	0.86072084	0.94335071	8.44E-06	0.99992186	-7.04E-05	-1.721384378	0.085181402	7.04152E-05
sex_FEMALE	0.90094834	0.86058831	0.94320119	8.17E-06	1.00007811	7.04E-05	1.720474765	0.085346482	7.03708E-05
depression_severity_Mild	0.90095725	0.86059445	0.9432131	8.19E-06	1.00006822	6.15E-05	1.502689627	0.13291941	6.1465E-05
race_ethnicity_Black_or_African_American_Non_Hispanic	0.90095733	0.86059623	0.94321132	8.19E-06	1.00006813	6.14E-05	1.500733251	0.133424881	6.13837E-05
cdm_name_ACT	0.90107583	0.86071511	0.94332915	8.38E-06	0.99993661	-5.71E-05	-1.396550241	0.162549168	5.71221E-05
region_8	0.90107145	0.86070615	0.9433298	8.39E-06	0.99994147	-5.27E-05	-1.28936012	0.197273213	5.27408E-05
race_ethnicity_Native_Hawaiian_or_Other_Pacific_Islander_Non_Hispanic	0.90098114	0.86062089	0.94323414	8.22E-06	1.00004171	3.76E-05	0.918690916	0.358257481	3.75763E-05
DIABETESUNCOMPLICATED_before_or_day_of_covid_indicator	0.90105244	0.86068596	0.94331212	8.37E-06	0.99996257	-3.37E-05	-0.824503768	0.40965357	3.37265E-05
race_ethnicity_Unknown	0.90104989	0.86068967	0.94330272	8.33E-06	0.99996539	-3.12E-05	-0.762352045	0.445850097	3.11817E-05
DIABETESCOMPLICATED_before_or_day_of_covid_indicator	0.90103523	0.86066538	0.94329864	8.35E-06	0.99998167	-1.65E-05	-0.403680557	0.686447724	1.65133E-05
region_6	0.90101275	0.86064979	0.94326867	8.29E-06	1.00000661	5.96E-06	0.145667061	0.884184254	5.95828E-06
sex_MALE	0.90102452	0.86065828	0.94328399	8.32E-06	0.99999356	-5.80E-06	-0.14190895	0.887151953	5.8048E-06
Grouped covariates									
healthcare utilization (number of visits before covid, visits per month before covid, post-COVID observation indicator)	0.92972829	0.88813404	0.97327054	0.0018072	0.96912046	-0.0287096	-691.263124	0	0.028709578
general health and comorbidities (BMI_max_observed_or_calculated_before_or_day_of_covid, CHRONICLUNGDISEASE_before_or_day_of_covid_indicator, DIABETESUNCOMPLICATED_before_or_day_of_covid_indicator, DIABETESCOMPLICATED_before_or_day_of_covid_indicator, OBESITY_before_or_day_of_covid_indicator, OTHERIMMUNOCOMPROMISED_before_or_day_of_covid_indicator, TOBACCOSMOKER_before_or_day_of_covid_indicator, SYSTEMICCORTICOSTEROIDS_before_or_day_of_covid_indicator, HYPERTENSION_before_or_day_of_covid_indicator, number_of_COVID_vaccine_doses_before_or_day_of_covid)	0.92030803	0.87930982	0.9632178	0.00035451	0.97904037	-0.0192893	-467.885442	0	0.01928932

Appendix 2. Nonparametric sensitivity analysis.

Our nonparametric sensitivity analysis contextualizes these relationships and indicates that, for our observed, adjusted estimate to be a Type 1 error, we would need to have 0.65 units of bias, where one unit refers to the difference between adjusted and unadjusted estimates (33). The qualitative shift in our observed measure of association following multivariate adjustment underscores the need for additional research in this area.
